# The impact of the COVID-19 lockdown on HIV care in 65 South African primary care clinics: an interrupted time series analysis

**DOI:** 10.1016/S2352-3018(20)30359-3

**Published:** 2021-02-04

**Authors:** Jienchi Dorward, Thokozani Khubone, Kelly Gate, Hope Ngobese, Yukteshwar Sookrajh, Siyabonga Mkhize, Aslam Jeewa, Christian Bottomley, Lara Lewis, Kathy Baisley, Christopher C Butler, Nomakhosi Gxagxisa, Nigel Garrett

**Affiliations:** a Centre for the AIDS Programme of Research in South Africa (CAPRISA), University of KwaZulu-Natal, Durban, KwaZulu-Natal, South Africa; b Discipline of Public Health Medicine, School of Nursing and Public Health, University of KwaZulu-Natal, Durban, KwaZulu-Natal, South Africa; c Department of Family Medicine, University of KwaZulu-Natal, Durban, KwaZulu-Natal, South Africa; d Nuffield Department of Primary Care Health Sciences, University of Oxford, Oxford, Oxfordshire, UK; e eThekwini Municipality Health Unit, eThekwini Municipality, Durban KwaZulu-Natal, South Africa; f Bethesda Hospital, uMkhanyakude District, KwaZulu-Natal, South Africa; g London School of Hygiene & Tropical Medicine, London, UK

## Abstract

**Background:**

The effect of the COVID-19 pandemic on HIV outcomes in low-income and middle-income countries is poorly described. We aimed to measure the impact of the 2020 national COVID-19 lockdown on HIV testing and treatment in KwaZulu-Natal, South Africa, where 1·7 million people are living with HIV.

**Methods:**

In this interrupted time series analysis, we analysed anonymised programmatic data from 65 primary care clinics in KwaZulu-Natal province, South Africa. We included data from people testing for HIV, initiating antiretroviral therapy (ART), and collecting ART at participating clinics during the study period, with no age restrictions. We used descriptive statistics to summarise demographic and clinical data, and present crude summaries of the main outcomes of numbers of HIV tests per month, ART initiations per week, and ART collection visits per week, before and after the national lockdown that began on March 27, 2020. We used Poisson segmented regression models to estimate the immediate impact of the lockdown on these outcomes, as well as post-lockdown trends.

**Findings:**

Between Jan 1, 2018, and July 31, 2020, we recorded 1 315 439 HIV tests. Between Jan 1, 2018, and June 15, 2020, we recorded 71 142 ART initiations and 2 319 992 ART collection visits. We recorded a median of 41 926 HIV tests per month before lockdown (January, 2018, to March, 2020; IQR 37 838–51 069) and a median of 38 911 HIV tests per month after lockdown (April, 2020, to July, 2020; IQR 32 699–42 756). In the Poisson regression model, taking into account long-term trends, lockdown was associated with an estimated 47·6% decrease in HIV testing in April, 2020 (incidence rate ratio [IRR] 0·524, 95% CI 0·446–0·615). ART initiations decreased from a median of 571 per week before lockdown (IQR 498–678), to 375 per week after lockdown (331–399), with an estimated 46·2% decrease in the Poisson regression model in the first week of lockdown (March 30, 2020, to April 5, 2020; IRR 0·538, 0·459–0·630). There was no marked change in the number of ART collection visits (median 18 519 visits per week before lockdown [IQR 17 074–19 922] *vs* 17 863 visits per week after lockdown [17 509–18 995]; estimated effect in the first week of lockdown IRR 0·932, 95% CI 0·794–1·093). As restrictions eased, HIV testing and ART initiations gradually improved towards pre-lockdown levels (slope change 1·183/month, 95% CI 1·113–1·256 for HIV testing; 1·156/month, 1·085–1·230 for ART initiations).

**Interpretation:**

ART provision was generally maintained during the 2020 COVID-19 lockdown, but HIV testing and ART initiations were heavily impacted. Strategies to increase testing and treatment initiation should be implemented.

**Funding:**

Wellcome Trust, Africa Oxford Initiative.

## Introduction

The number of deaths from COVID-19 continues to rise globally, but data quantifying the effect of the pandemic on other health conditions are scarce, especially in low-income and middle-income countries. Interruption of supply chains, diversion of resources, and overwhelmed health systems could have severe collateral effects on existing public health programmes.^1^, ^2^, ^3^, ^4^ Furthermore, COVID-19 control measures, such as stay-at-home orders, or lockdowns, might limit access to health-care services, further jeopardising broader public health goals.^3^, ^5^, ^6^

In Africa, the impact on HIV services is of particular concern. Despite improvement in HIV prevention, testing, and treatment, HIV/AIDS remains one of the leading causes of mortality, with more than 400 000 deaths on the continent in 2019.^7^ Of the estimated 25·8 million people in Africa living with HIV in 2019, 4·3 million were not diagnosed, and a further 3·4 million were not receiving antiretroviral therapy (ART).^7^ Modelling studies of disruptions to HIV programmes by the COVID-19 pandemic estimate that interruptions in ART would have the largest effect on HIV-related mortality.^8^, ^9^ In a worst case scenario, interruption of ART for 6 months for 50% of patients would result in 296 000 excess HIV-related deaths.^8^

Such scenarios are of particular concern in the province of KwaZulu-Natal, in South Africa, which is the country most heavily affected by both COVID-19 and HIV in Africa. 493 183 cases of COVID-19 had been confirmed in South Africa by July 31, 2020,^10^ and in 2019 an estimated 7·5 million people were living with HIV in the country. An estimated 1·7 million people are living with HIV in KwaZulu-Natal, a prevalence of 27% in adults aged 15–49 years.^11^ 76 706 cases of COVID-19 had been confirmed in KwaZulu-Natal by July 31, 2020, making it the third most COVID-19 affected province in South Africa.^10^ The largest urban area in KwaZulu-Natal is the eThekwini Metropolitan Municipality, which has a population of approximately 3·7 million and includes the city of Durban.^12^ South Africa announced a national lockdown on March 23, 2020, which was implemented on March 27.^6^ Starting at level 5, the lockdown was one of the most severe globally, with restrictions on movement and cancellation of public transport, although travel to receive health care was allowed.^6^ The lockdown was eased to level 4 on May 1, 2020, when public transport was allowed,^6^ and to level 3 on June 1, 2020, which allowed some economic activity to resume.

**Research in context**

**Evidence before this study**

The COVID-19 pandemic could greatly affect HIV programmes in low-income and middle-income countries. Modelling studies have suggested that disruptions to antiretroviral therapy (ART) provision would have the worst consequences, with a 6-month interruption in treatment for half of people who receive ART, leading to nearly 300 000 excess HIV deaths in sub-Saharan Africa. However, whether such high levels of disruption have occurred is not clear. We searched PubMed for the terms (COVID-19 OR SARS-CoV-2) AND (HIV OR AIDS) AND (LMIC OR low income country OR middle income country OR Asia OR Africa OR Latin America) AND (lockdown OR lock-down OR curfew OR impact OR shelter OR restriction) from inception until Oct 16, 2020. We found four small studies that provided quantitative data comparing HIV care outcomes before and after COVID-19 lockdowns. A single site cohort study of pre-exposure prophylaxis in 455 pregnant women in South Africa found an increase in missed visits after lockdown. An interrupted time series analysis from 11 clinics in rural South Africa found a 20% increase in HIV-related primary care visits after lockdown implementation, and two studies from Kenya described a decrease in numbers of HIV tests in the first month of lockdown, compared with the previous 3 months.

**Added value of this study**

We contribute new evidence of the impact of the COVID-19 lockdown on HIV care in KwaZulu-Natal, South Africa, which has the largest ART programme in the world, and had one of the strictest lockdowns in Africa. We analysed a large dataset from urban and rural primary care clinics between Jan 1, 2018, and July 31, 2020, and used interrupted time series analysis to account for longer-term trends. HIV testing and ART initiations decreased substantially when lockdown was implemented, but ART collection visits decreased only slightly.

**Implications of all the available evidence**

ART provision was largely maintained during the South African lockdown, while HIV testing and ART initiations were more heavily affected. After lockdown, and in any future COVID-19 restrictions, strategies to catch up with HIV testing and increase ART initiation should be implemented, alongside efforts to maintain treatment provision.

The impact of lockdown measures in South Africa and other African countries on HIV programmes is not clear. An interrupted time series analysis^13^ of data from 11 rural clinics in South Africa found a 20% increase in HIV-related primary care visits after lockdown implementation. By contrast, two small studies in Kenya^14^, ^15^ described 16–30% decreases in HIV testing after lockdown. However, these analyses were from few sites and might be biased by long-term trends that were not accounted for.

We aimed to quantify the impact of COVID-19 lockdown in South Africa on key components of HIV care, namely HIV testing, ART initiation, and retention in HIV care, which was measured using ART collection visits and missed visits.

## Methods

### Study design

We did an interrupted time series analysis of routinely collected data from 65 public sector, primary care clinics in KwaZulu-Natal, South Africa. We used data from 56 urban clinics run by the eThekwini Municipality Health Unit, which represents approximately 60% of all public clinics in the Metro. We also used data from all nine fixed and mobile primary care clinics overseen by Bethesda Hospital, a district hospital in the rural uMkhanyakude District in northern KwaZulu-Natal. ART is provided free of charge at all these clinics, and they remained open during the South African national lockdown. Before and throughout the lockdown, there were no reports of ART stockouts in the study clinics, and public health messaging highlighted the importance of ART for all people living with HIV.^16^

### Participants

We included data from people testing for HIV, initiating ART, and collecting ART at participating clinics during the study period, with no age restrictions. For ART initiations, we excluded patients who were already receiving ART and were transferring into care from another clinic. For HIV testing, data were available from Jan 1, 2018, to July 31, 2020, for all other outcome variables data were available until June 15, 2020. We chose not to use data before January, 2018, as earlier trends might have been influenced by changes in ART initiation criteria and the implementation of universal test and treat in South Africa at the end of 2016.

### Data sources and data management

In the South African public sector, data on the number of HIV tests done per month at each clinic are routinely recorded in the District Health Information System^12^ and reported by gender and age group. For patients initiating and receiving ART, data on demographics, clinical status, and clinic visits are routinely recorded in an electronic register^17^ that is compared monthly against clinic registers and a subset of clinical charts. For this analysis, anonymised patient-level data were analysed and aggregated into weekly counts of ART initiations and ART collection visits. During the COVID-19 lockdown, clinical staff, data capturers, and health information system managers were classed as essential workers. This designation meant that transport arrangements were made to enable attendance at work, minimising disruptions to data entry and data capture.

### Outcomes

The primary outcomes were HIV tests per month, ART initiations per week, and ART collection visits per week at participating clinics during the study period. The number of weekly ART collection visits was a function of when visits were scheduled, and whether patients attended these scheduled visits. Because visit scheduling might also have changed during lockdown, we assessed a secondary outcome of missed ART collection visits per week. For the missed visits variable, we used the date of next scheduled ART collection visit to calculate visits that were missed by more than 2 weeks, as defined by South African guidelines.^18^ Patients who attended earlier than their next scheduled visit were not defined as missing a visit, and we restricted the period of interest to end 2 weeks before the last day of data collection, because by definition a missed visit required no visit in the next 2 weeks. We stratified the outcome data by gender and by rural or urban district (according to clinic location).

### Statistical analysis

We used descriptive statistics to summarise demographic and clinical data, and present crude summaries of outcomes before and after lockdown. We did not summarise HIV testing data by age, because different age categories were used for recording during the study period. We did interrupted time series segmented regression analyses by fitting a Poisson regression model with Newey–West standard errors^19^, ^20^ to account for autocorrelation and heteroskedasticity. The model included a time variable, a dummy lockdown variable indicating pre-lockdown and post-lockdown, and an interaction term between time and the lockdown variable. This approach takes account of pre-lockdown trends and allows estimation of the effect of lockdown at various timepoints by centring time at that timepoint. We used the model to estimate the immediate impact of lockdown and the effect of the lockdown by the end of the study period. We estimated the post-lockdown trend for each outcome by adding together the coefficients associated with time and the time–lockdown interaction. We did analyses for ART initiations and ART collection visits by weekly counts, but we present trends of these outcomes by monthly periods to facilitate comparisons with monthly HIV testing trends. We built separate models by gender, age, and rural and urban group. To account for seasonal changes in clinic activity (eg, holiday periods when clinics remain open but visits are typically fewer), we did a sensitivity analysis with two pairs of sine and cosine terms (Fourier terms) included in the model. We also did a sensitivity analysis of trends in positive HIV tests. We analysed data using R 4.0 (R Foundation for Statistical Computing, Vienna, Austria; appendix).

### Ethical approval

This work was approved by University of Kwazulu-Natal Biomedical Research Ethics Committee (BE646/17), the KwaZulu-Natal Department of Health's Provincial Health Research Ethics Committee (KZ_201807_021), the eThekwini Municipality Health Unit, and the Bethesda Hospital Ethics Committee, with a waiver for informed consent for analysis of anonymised, routinely collected data.

### Role of the funding source

The funders had no role in the study design, data collection, analysis, interpretation, or writing of the paper.

## Results

Between Jan 1, 2018, and July 31, 2020, 1 315 439 HIV tests were done at all participating clinics. Between Jan 1, 2018, and June 15, 2020, 71 142 people started ART, and 235 719 people attended 2 319 992 ART collection visits (median 9 visits per person, IQR 5–14). Between Jan 1, 2018, and June 1, 2020, 339 474 ART collection visits were missed. Demographic details are presented in table 1. 65·4–68·1% of outcomes were recorded in women, reflecting the higher prevalence of HIV among women in South Africa,^11^ and 91·8–96·7% of outcomes occurred at urban clinics. Pre-lockdown, December, 2018 and 2019 had the lowest counts of HIV tests, ART initiations, ART collection visits, and missed visits (figure).

**Table 1:** Demographics of people who had an HIV test, initiated ART, or collected ART at 65 clinics in KwaZulu-Natal, South Africa

		HIV tests*	**ART initiations**†	**ART collection visits**†	**Missed ART collection visits**‡
Total in study period		1 315 439	71 142	2 319 992	339 474
Median age, years (IQR)§		NA	32 (27–39)	37 (31–45)	36 (30–43)
By age group§					
	0–14 years	NA	891 (1·2%)	48 440 (2·1%)	7112 (2·1%)
	15–24 years	NA	9734 (13·7%)	135 649 (5·9%)	26 249 (7·7%)
	25–49 years	NA	55 840 (78·5%)	1 785 187 (76·9%)	265 408 (78·2%)
	≥50 years	NA	4741 (6·7%)	457 647 (15·1%)	40 705 (12·0%)
Gender					
	Female	861 265 (65·5%)	46 520 (65·4%)	1 580 202 (68·1%)	224 799 (66·2%)
	Male	454 174 (34·5%)	24 622 (34·6%)	739 790 (31·9%)	114 675 (33·8%)
District of clinic					
	Urban	1 269 811 (96·5%)	68 821 (96·7%)	2 216 761 (95·6%)	311 746 (91·8%)
	Rural	45 628 (3·5%)	2321 (3·3%)	103 231 (4·4%)	27 728 (8·2%)

Data are n (%) unless otherwise specified. ART=antiretroviral therapy.

* Jan 1, 2018, to July 31, 2020.

† Jan 1, 2018, to June 15, 2020.

‡ Jan 1, 2018, to June 1, 2020.

§ Age in routine HIV testing data was recorded using varying age categories during the study period and is therefore not available.

**Figure:**
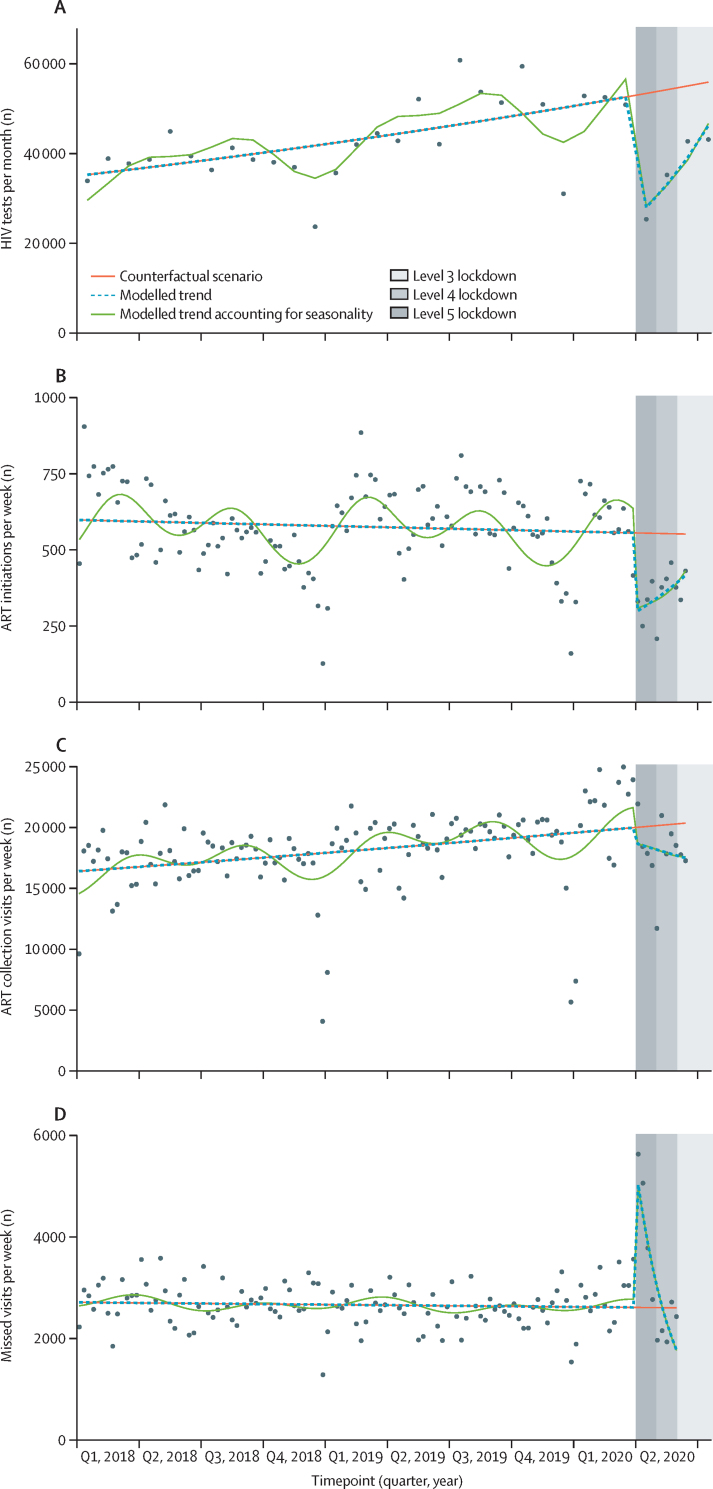
Poisson segmented regression analyses of HIV testing, ART initiations, ART collection visits, and missed visits, before and after the COVID-19 lockdown in 65 clinics in KwaZulu-Natal, South Africa (A) HIV tests per month. (B) ART initiations per week. (C) ART collection visits per week. (D) Missed visits per week. ART=antiretroviral therapy.

In the 27 months before the lockdown on March 27, 2020, a median of 41 926 HIV tests were done per month (IQR 37 838–51 069), compared with 38 911 tests per month (32 699–42 756) in the 4 months after lockdown (April, 2020–July, 2020). Before lockdown, a median of 2691 (2425–2906) of these HIV tests were positive per month, compared with 1638 per month (1568–1662) after lockdown. The median proportion of positive tests was 6·1% (IQR 5·4–7·0%) before lockdown and 4·3% (4·0–4·8%) after lockdown. The Poisson segmented regression analysis showed a 47·6% decrease in HIV testing in the first month of lockdown (April, 2020) (incidence rate ratio [IRR] 0·524, 95% CI 0·446–0·615; figure, table 2). After April, 2020, HIV testing increased by a trend of 18·3% per month (IRR 1·183, 95% CI 1·113–1·256), reaching 82·7% of pre-lockdown levels (0·827, 95% CI 0·704–0·972) by July, 2020. Findings were similar between women and men (table 2), and in a sensitivity analysis of positive HIV tests (appendix p 1). In the urban clinics, the decrease in HIV testing at lockdown was larger than in the rural clinics (IRR 0·515, 95% CI 0·436–0·610 in the urban centres *vs* 0·823, 0·681–0·994 in the rural centres; table 2).

**Table 2:** Poisson segmented regression models of the impact of COVID-19 lockdown on HIV services at 65 clinics in KwaZulu-Natal, South Africa

		**Incidence rate ratio at lockdown**	**Incidence rate ratio at study end**	Pre-lockdown trend*	Post-lockdown trend*
**HIV testing**†					
Overall		0·524 (0·446–0·615)	0·827 (0·704–0·972)	1·015 (1·009–1·022)	1·183 (1·113–1·256)
Gender					
	Women	0·563 (0·480–0·661)	0·865 (0·741–1·010)	1·013 (1·007–1·019)	1·169 (1·100–1·247)
	Men	0·456 (0·387–0·537)	0·761 (0·638–0·907)	1·020 (1·013–1·028)	1·210 (1·147–1·277)
District					
	Urban	0·515 (0·436–0·610)	0·827 (0·700–0·978)	1·016 (1·010–1·023)	1·190 (1·115–1·270)
	Rural	0·823 (0·681–0·994)	0·785 (0·639–0·965)	0·996 (0·985–1·006)	0·980 (0·922–1·042)
**ART initiation**‡					
Overall		0·538 (0·459–0·630)	0·753 (0·637–0·890)	0·996 (0·991–1·004)	1·156 (1·085–1·230)
Gender					
	Women	0·495 (0·417–0·588)	0·801 (0·651–0·985)	0·996 (0·987–1·004)	1·225 (1·118–1·341)
	Men	0·616 (0·513–0·740)	0·675 (0·571–0·797)	1·000 (0·996–1·009)	1·040 (0·957–1·132)
Age, years					
	0–14	0·370 (0·153–0·896)	0·544 (0·270–1·096)	0·996 (0·987–1·004)	1·175 (0·640–2·164)
	15–24	0·400 (0·334–0·479)	0·772 (0·640–0·932)	1·022 (1·013–1·031)	1·363 (1·256–1·476)
	25–49	0·577 (0·492–0·676)	0·739 (0·624–0·876)	0·991 (0·987–1·000)	1·108 (1·040–1·180)
	≥50	0·481 (0·399–0·579)	0·796 (0·664–0·953)	0·991 (0·983–1·000)	1·230 (1·161–1·335)
District					
	Urban	0·536 (0·458–0·628)	0·750 (0·631–0·892)	0·996 (0·991–1·004)	1·156 (1·080–1·230)
	Rural	0·588 (0·365–0·947)	0·863 (0·625–1·191)	0·987 (0·983–0·996)	1·166 (0·869–1·560)
**ART collection visits**‡					
Overall		0·932 (0·794–1·093)	0·859 (0·747–0·989)	1·009 (1·000–1·013)	0·974 (0·900–1·053)
Gender					
	Women	0·916 (0·780–1·075)	0·865 (0·753–0·994)	1·009 (1·000–1·013)	0·983 (0·908–1·062)
	Men	0·965 (0·823–1·131)	0·847 (0·733–0·978)	1·009 (1·004–1·013)	0·953 (0·880–1·031)
Age, years					
	0–14	0·908 (0·790–1·043)	0·780 (0·703–0·866)	1·013 (1·009–1·017)	0·949 (0·888–1·017)
	15–24	0·875 (0·766–1·001)	0·851 (0·753–0·960)	1·026 (1·017–1·031)	1·013 (0·957–1·071)
	25–49	0·933 (0·796–1·094)	0·865 (0·751–0·995)	1·009 (1·000–1·013)	0·974 (0·900–1·053)
	≥50	0·946 (0·787–1·137)	0·836 (0·710–0·984)	1·004 (1·000–1·009)	0·949 (0·861–1·049)
District					
	Urban	0·926 (0·786–1·090)	0·850 (0·736–0·982)	1·009 (1·004–1·013)	0·970 (0·896–1·053)
	Rural	1·065 (0·969–1·171)	1·080 (0·985–1·185)	0·996 (0·991–1·000)	1·000 (0·957–1·044)
**Missed ART collection visits**‡					
Overall		1·926 (1·585–2·341)	0·682 (0·455–1·021)	1·000 (0·996–1·004)	0·569 (0·432–0·747)
Gender					
	Women	2·045 (1·686–2·481)	0·691 (0·454–1·052)	0·996 (0·991–1·000)	0·555 (0·419–0·734)
	Men	1·710 (1·399–2·090)	0·664 (0·457–0·965)	1·000 (0·996–1·004)	0·601 (0·463–0·779)
Age, years					
	0–14	1·568 (1·325–1·856)	0·555 (0·429–0·717)	1·004 (1·000–1·009)	0·572 (0·472–0·690)
	15–24	1·682 (1·413–2·001)	0·554 (0·374–0·820)	1·022 (1·017–1·026)	0·561 (0·430–0·727)
	25–49	1·947 (1·594–2·378)	0·685 (0·451–1·042)	1·000 (0·996–1·000)	0·566 (0·428–0·751)
	≥50	2·030 (1·665–2·474)	0·782 (0·552–1·109)	0·987 (0·983–0·991)	0·589 (0·463–0·751)
District					
	Urban	1·991 (1·621–2·446)	0·677 (0·433–1·057)	1·000 (0·996–1·004)	0·555 (0·412–0·751)
	Rural	1·274 (1·111–1·460)	0·745 (0·636–0·872)	1·004 (0·996–1·009)	0·751 (0·674–0·834)

Data are incidence rate ratio (95% CI) or trend (95% CI). ART=antiretroviral therapy.

* Slope change per month

† Autocorrelation addressed using Newey–West standard errors to calculate CI, with lag up to 2.

‡ Autocorrelation addressed using Newey–West standard errors to calculate CI, with lag up to 3.

In the 117 weeks before lockdown, a median of 571 ART initiations were done per week (IQR 498–678), compared with 375 per week (331–399) in the 11 weeks after implementation of lockdown. The segmented regression analysis showed a 46·2% decrease in ART initiations in the first full week of lockdown (March 30, 2020, to April 5, 2020; IRR 0·538, 95% CI 0·459 −0·630; figure, table 2). Thereafter, ART initiations gradually recovered by a trend of 15·6% per month (IRR 1·156, 95% CI 1·085–1·230; trend presented by month to allow comparisons with monthly HIV testing trends), reaching 75·3% of pre-lockdown levels (IRR 0·753, 95% CI 0·637–0·890) by mid-June, 2020. This recovery in ART initiations occurred mainly in women (IRR 1·225 per month, 95% CI 1·118–1·341), whereas ART initiations remained low in men (1·040 per month, 0·957–1·132). The effect of lockdown on ART initiations was similar across age groups, and rural and urban clinics (table 2).

Patients attended a median of 18 519 ART collection visits per week before lockdown (IQR 17 074–19 922), compared with 17 863 per week (17 509–18 995) after implementation of lockdown. The segmented regression analysis showed weak evidence of a small decrease in the number of ART collection visits in the first full week of lockdown (IRR of lockdown effect 0·932, 95% CI 0·794–1·093; figure, table 2). After lockdown, the number of ART collections per month remained broadly constant (IRR 0·974, 95% CI 0·900–1·053), but by the end of data collection in mid-June, there was some evidence to suggest that ART collections visits were lower than pre-lockdown levels (IRR 0·859, 95% CI 0·747–0·989%). Results were similar between men and women, age groups, and for rural and urban clinics (table 2). In post-hoc analyses, we added an indicator term for the 4 weeks between the date of the first confirmed case of severe acute respiratory syndrome coronavirus 2 (SARS-CoV-2) in South Africa and the start of the South African lockdown. The number of ART collection visits were higher in the 4 weeks between SARS-CoV-2 identification and lockdown (IRR 1·233, 95% CI 1·113–1·366), even when taking seasonality into account (1·165, 1·042–1·303). We also found that the number of early visits, in which people attended more than 7 days before their scheduled visit date, increased in this pre-lockdown period (1·271, 1·110–1·456).

Before lockdown, a median of 2637 visits per week were missed (IQR 2420–2938), compared with 2714 per week after lockdown (2151–3777; figure). The Poisson regression model showed a large increase in missed visits in the first week of lockdown (IRR 1·926, 95% CI 1·550–2·394). Thereafter, missed visits decreased rapidly as lockdown continued (0·569 per month, 0·432–0·747), and returned to pre-lockdown levels by the end of May, 2020, when the lockdown moved from level 5 to level 4 (1·006, 0·794–1·276; figure). Of the 19 183 people who missed a visit by 2 weeks during the level 5 lockdown period, 11 259 (58·7%) subsequently collected within the next month (ie, 42 days after their scheduled visit). We did not observe any marked differences in results between men and women or in different age groups. In the rural clinics, the increase in missed visits in the first week of lockdown was much less marked (IRR 1·274, 95% CI 1·076–1·509) than in the urban clinics (1·991, 1·584–2·503; table 2).

After adding two Fourier terms to account for seasonality, our findings were similar to those when seasonality was not included (table 3, figure). We also did a post-hoc analysis among people referred into a community ART delivery programme.^21^ Community ART collections are not recorded as clinic visits, and so difficulties in collecting treatment from community pick-up points during lockdown could have led to an increase in ART collection visits at study clinics. We found no evidence of an increase in study clinic collection visits among people referred into the community ART delivery programme. The number of unscheduled clinic visits in this population decreased in the first week of lockdown (IRR 0·732, 95% CI 0·551–0·974), and was similar to pre-lockdown levels by the end of the study period (1·163, 0·973–1·391).

**Table 3:** Sensitivity analyses taking account of seasonality using two Fourier pairs in Poisson segmented regression models of the impact of COVID-19 lockdown on HIV services in KwaZulu-Natal, South Africa

	**Incidence rate ratio at lockdown**	**Incidence rate ratio at study end**	Pre-lockdown trend*	Post-lockdown trend*
HIV testing†	0·475 (0·404–0·559)	0·741 (0·631–0·872)	1·018 (1·012–1·023)	1·180 (1·090–1·279)
ART initiation‡	0·496 (0·411–0·598)	0·798 (0·645–0·987)	1·000 (0·991–1·004)	1·225 (1·113–1·352)
ART collection visits‡	0·859 (0·733–1·007)	0·852 (0·745–0·975)	1·009 (1·004–1·013)	1·004 (0·924–1·090)
Missed ART collection visits‡	1·812 (1·494–2·197)	0·693 (0·459–1·048)	1·000 (0·996–1·004)	0·595 (0·451–0·783)

Data are rate (95% CI) or trend (95% CI). ART=antiretroviral therapy.

* Slope change per month.

† Autocorrelation addressed using Newey–West standard errors to calculate CI, with lag up to 2.

‡ Autocorrelation addressed using Newey–West standard errors to calculate CI, with lag up to 3.

## Discussion

We present data from a large clinic population in KwaZulu-Natal, South Africa, that show an almost 50% decrease in HIV testing and ART initiations at the beginning of the COVID-19 lockdown, with a gradual improvement over the next 3 months towards pre-lockdown levels. ART collection visits decreased slightly and missed ART collection visits increased for a short time. These trends did not differ markedly by age or gender, apart from ART initiations, which remained low among men but gradually improved among women. Overall, the impact of the lockdown tended to be less marked in the rural clinics. These findings suggest that HIV services were generally maintained for people already receiving ART. However, engaging new people into care (through HIV testing and subsequent treatment initiation) was impeded by the lockdown, particularly in urban clinics.

To date, there are few published data from low-income and middle-income countries that quantify the effect of COVID-19 lockdowns on HIV services and patient-related outcomes along the HIV care cascade. The studies that have been published were small, and unlike our analysis, they do not account for long-term trends or seasonal variations, which might have influenced outcomes. A single-site, pre-exposure prophylaxis study^22^ of 455 pregnant women in South Africa reported that 34% of patients missed visits before lockdown, increasing to 57% after lockdown (odds ratio 2·36, 95% CI 1·73–3·16). In keeping with our findings, two descriptive analyses from small studies in Kenya (two sites^14^ and three sites^15^) reported that 15–30% fewer HIV tests were done in April, 2020, than were done per month in January to March, 2020.^14^, ^15^ National laboratory data^23^ from South Africa comparing the 2 months pre-lockdown with the first month of lockdown showed decreases of 33% in CD4 cell count (usually done at HIV diagnosis or ART initiation) and 22% in viral load testing (usually done for ART monitoring). However, decreased HIV viral load PCR testing could reflect changes in laboratory system capacity due to increased SARS-CoV-2 PCR testing, rather than changes in patient clinic attendance and ART provision.

An interrupted time series analysis^13^ in 11 primary care clinics in rural KwaZulu-Natal found no difference in the number of overall clinic visits, but did show a 20% increase in HIV-related visits immediately after lockdown implementation. The study did not distinguish between HIV testing, ART initiation, or ART collection visits, although the authors hypothesise that the increase in use of HIV services reflected a rush to collect ART in anticipation of further restrictions or drug shortages. In our study, we also found that the COVID-19 lockdown affected the rural clinics less than clinics in urban areas, where lockdown restrictions might have been more heavily enforced. Furthermore, some people who had migrated to urban areas for work might have moved back to rural areas during the lockdown.

Why were HIV testing and ART initiation most affected? A qualitative study in rural Uganda,^24^ and anecdotal reports from studies in Kenya,^14^, ^15^ suggest that drops in testing could be due to a paucity of personal protective equipment and space for physical distancing in clinics, as well as reduced clinic opening times and staff being redeployed from HIV testing to COVID-19 response activities.^14^, ^24^ In South Africa, 28 000 HIV community health-care workers were diverted from HIV outreach to COVID-19 symptom screening,^25^ which might have led to fewer referrals to clinics for HIV testing. People without established patterns of engagement in care and regular clinic attendance to collect treatment might also have been less likely to overcome the challenges involved in attending clinics during lockdown. These include increased costs, transport difficulties, the potential need to provide proof of the reason for travel, paucity of resources, and fear of contracting SARS-CoV-2 infection at clinics.^14^, ^15^, ^24^ The decrease in HIV testing, positive HIV tests, and ART initiations were broadly similar, suggesting that the decrease in ART initiations was largely due to decreased testing, rather than additional attrition in the cascade between testing HIV positive and initiating ART.

The lockdown did cause some disruption to ART collections, with a short increase in missed ART collection visits in the first month of lockdown. However, collections rapidly returned to pre-lockdown levels, and most people who missed a visit did attend in the next month, meaning that overall ART collection visits did not decrease drastically in the study clinics. These findings are similar to findings from a survey^26^ of 301 patients in a community ART delivery programme in urban KwaZulu-Natal, of whom only 8% reported delaying ART collection during the lockdown,^26^ despite 34% reporting increased travel times or costs to collect ART and 51% reporting long waiting times to collect treatment.^26^ ART collection might have been prioritised by patients already engaged in HIV care and aware of the importance of maintaining high adherence. Similar to the results of the study from rural KwaZulu-Natal,^13^ we found a pre-lockdown increase in ART collection visits, suggesting that people were stocking up in preparation for potential disruptions. Clinics were also able to facilitate ART provision through strategies such as multi-month prescribing and differentiated service delivery programmes.^16^, ^27^, ^28^ We plan further analyses to quantify how these strategies were implemented, as they might account for the slight decrease in ART collection visits after lockdown. For this analysis, any unmeasured increase in multi-month prescribing or community ART delivery would bias our results towards larger decreases in clinic ART collection visits after lockdown, rather than masking a substantial decrease in visits. We also found no evidence of an increase in people returning early to study clinics instead of collecting in the community ART delivery programme during lockdown.

Strengths of our study include the large number of routine public sector clinics from both urban and rural settings that are likely to reflect aspects of HIV programmes across southern and eastern Africa; although South Africa's large ART programme might be better resourced and hence more resilient to COVID-19 impacts.^29^ Our use of long-term routine data takes into account underlying trends in HIV testing and ART use, and allows more accurate quantification of the effect of the COVID-19 lockdown. However, due to the format of HIV testing data, we were not able to assess HIV testing by weekly counts, which would have allowed a more detailed assessment of shorter-term trends. We were also unable to assess HIV viral load outcomes due to the time taken for these results to be entered into the records system.

Our findings suggest that, in one of the regions most affected by both HIV and COVID-19, the worst modelled scenarios of the impact of COVID-19 on HIV are unlikely to play out. We did not find evidence of large disruptions to ART provision, which is the main driver of morbidity and mortality in published models.^8^ Instead, efforts to continue providing treatment to people in the ART programme appear to have been largely successful. Although this evidence is reassuring, COVID-19-related disruptions to ART supply chains and future COVID-19 outbreaks and lockdowns still pose a threat to HIV programmes.^30^ Furthermore, our findings suggest that people who are not yet in HIV care were most affected by the lockdown. As countries in Africa consolidate health systems after the first wave of COVID-19, and manage potential second waves, efforts to catch-up with HIV testing and initiation of ART should be prioritised. Integrating HIV and SARS-CoV-2 testing programmes could be beneficial,^31^ and WHO and other international organisations are now advocating for an increased focus on HIV self-testing.^28^ Innovative strategies to facilitate access to treatment are also required, such as home and community-based ART initiation.^32^ Research into HIV service provision in other settings, and the factors that impeded HIV testing and treatment initiation during the COVID-19 lockdown, is needed to inform public health responses to future COVID-19 outbreaks.

In conclusion, engagement of people in HIV care in South African primary care clinics through HIV testing and treatment initiation was severely impacted by the COVID-19 lockdown, with a gradual recovery towards pre-lockdown levels as restrictions eased. There were no large changes in ART collection visits. Strategies to increase HIV testing and treatment initiation should be implemented to address the current and potential future outbreaks.
