# Supplementary material for: The impact of the COVID-19 lockdown on HIV care in 65 South African primary care clinics: an interrupted time series analysis
Source: Lancet HIV. 2021 Feb 4;8(3):e158–65. doi: 10.1016/S2352-3018(20)30359-3 (PMC8011055; doi:10.1016/S2352-3018(20)30359-3)
Supplement: Supplementary appendix [file mmc1.pdf]

THE LANCET
HIV

Supplementary appendix

This appendix formed part of the original submission and has been peer reviewed. We post it as supplied by the authors.

Supplement to: Dorward J, Khubone T, Gate K, et al. The impact of the COVID-19 lockdown on HIV care in 65 South African primary care clinics: an interrupted time series analysis. *Lancet HIV* 2021; published online Feb 4. http://dx.doi.org/10.1016/S2352-3018(20)30359-3.

Supplementary Material

Formula for Poisson regression models

Calculating the change in outcome at lockdown, no seasonality

$$\log(\mathrm{y}) = b_0 + b_1 t + b_2 x + b_3 x t$$

where y = mean outcome of interest, x = lockdown indicator and t = time in weeks since the start of the study.

Calculating the change in outcome at time T

To estimate the intervention at this timepoint, we centre t about T

$$\log(\mathrm{y}) = b_0 + b_1(t - T) + b_2 x + b_3 x(t - T)$$

Model including seasonality

$$\log(\text{mean inititiations}) = b_0 + b_1 t + b_2 x + b_3 x t + b_4 \sin\left(\frac{2\pi \mathrm{t}}{52}\right) + b_5 \cos\left(\frac{2\pi \mathrm{t}}{52}\right) + b_6 \sin\left(\frac{4\pi \mathrm{t}}{52}\right) + b_7 \cos\left(\frac{4\pi \mathrm{t}}{52}\right)$$

Table S1: Segmented Poisson regression models of the impact of COVID-19 lock-down on positive HIV tests at 65 clinics in KwaZulu-Natal, South Africa

	**Incidence rate ratio at lock-down (95% CI)**	**Incidence rate ratio at study end (95% CI)**	**Pre-lockdown trend[a] (95% CI)**	**Post-lockdown trend[a] (95% CI)**
Positive HIV test results [b]	0.611 (0.532-0.702)	0.739 (0.634-0.861)	0.993 (0.986-1.000)	1.057 (1.017-1.100)
Positive HIV test results, accounting for seasonality [b]	0.602 (0.511-0.710)	0.696 (0.571-0.848)	0.994 (0.988-1.001)	1.044(0.964-1.130)

[a] Slope change per month [b] Autocorrelation addressed using Newey-West standard errors to calculate confidence interval, with lag up to 2.

Sample R code for Poisson regression models, using ART initiations as an example

```
###############
## Install and load packages
###############
# install.packages("sandwich"); install.packages("tidyverse"); install.packages("tsModel")
library(sandwich); library(tidyverse); library(tsModel)

###############
### Centre time
###############

data$time_c <- data$time - 118  # Time centred at lockdown
data$time_e <- data$time - 128 # Time centred at end of the study period

###############
### Poisson regression model of ART initiations pre and post lockdown
###############

model_init <- glm(init_n ~ lock_d + time + lock_d:time_c, family = poisson, data)
summary(model_init)

## Look at residuals for autocorrelation

data$res1 <- residuals(model_init, type = "deviance")
ggplot(data = data, mapping = aes(x = time, y = res1,)) +
  geom_point(aes(colour = lock_d))
acf(data$res1)
pacf(data$res1)

## Calculate Newey-West standard errors using lag 3

est <- exp(c(coef(model_init)["lock_d:time_c"], coef(model_init)["lock_d"], coef(model_init)["time"]))
se1 <- sqrt(diag(NeweyWest(model_init, prewhite = F, lag = 3)))["lock_d:time_c"]
se2 <- sqrt(diag(NeweyWest(model_init, prewhite = F, lag = 3)))["lock_d"]
```

```
se3 <- sqrt(diag(NeweyWest(model_init, prewhite = F, lag = 3)))["time"]
lb <- est * exp(-1.96 * c(se1, se2, se3))
ub <- est * exp(1.96 * c(se1, se2, se3))
table <- cbind(round(est, digits = 3), round(lb, digits = 3), round(ub, digits = 3))
table

## Calculate post-lockdown trend and confidence intervals

V <- NeweyWest(model_init, prewhite = F, lag = 3)
se <- sqrt(V["time", "time"] + V["lock_d:time_c", "lock_d:time_c"]
        + 2 * V["time", "lock_d:time_c"])
slope_post <- sum(coef(model_init)[c("time", "lock_d:time_c")])
lower <- slope_post - 1.96 * se
upper <- slope_post + 1.96 * se
exp(cbind(slope_post, lower, upper))

## Estimate the impact of lockdown at the end of the study period

model_init_end <- glm(init_n ~ lock_d + time + lock_d:time_e, family = poisson, data)
summary(model_init_end)

##############
### Poisson regression model of ART initiations adjusting for seasonality
##############
# Specifying the number of sin and cosine pairs to include (2) and the interval (52 weeks)

model_seas <- glm(init_n ~ lock_d + time + lock_d:time_c + harmonic(wk, 2, 52),
            family = poisson, data)
summary(model_seas)
```
